# Application of high-dose tranexamic acid in the perioperative period: a narrative review

**DOI:** 10.3389/fphar.2025.1552511

**Published:** 2025-03-21

**Authors:** Yushan Duan, Xiaohong Wan, Yiming Ma, Weihua Zhu, Yue Yin, Qingqing Huang, Yuan Yang

**Affiliations:** Department of Critical Care Medicine, The Second Affiliated Hospital, Kunming Medical University, Kunming, China

**Keywords:** high-dose, tranexamic acid, perioperative period, blood loss, adverse reactions

## Abstract

**Objective:**

To investigate the efficacy and safety of high-dose tranexamic acid in different types of surgeries and provide a reference for clinical practice.

**Methods:**

We systematically searched PubMed, Cochrane Library, Science, Embase, and CNKI databases, from their inception to January 2025, to include representative literature related to high-dose tranexamic acid in the perioperative period for a thematic synthesis. The analysis focused on clinical evidence related to obstetric, cardiac, urologic, orthopedic, and spinal surgeries.

**Results:**

High-dose tranexamic acid markedly reduces blood loss and transfusion requirements in most types of surgery; however, the optimal dose varies by surgery type. Available studies have shown a favorable safety profile; however, some areas (e.g., cardiac surgery) still require careful monitoring for seizures and risk of thrombotic events.

**Conclusion:**

The clinical benefit of high-dose tranexamic acid should be assessed based on surgical characteristics and patient individualization. More multicenter studies are needed to clarify the dose-effect relationship and long-term safety.

## 1 Introduction

Tranexamic acid (TXA), a lysine derivative, competitively inhibits the transformation of plasminogen into plasmin, an enzyme responsible for fibrin clot breakdown. Since its introduction in the 1960s, TXA has been used in cases of menorrhagia and congenital bleeding disorders (Tengborn et al., 2015), with extensive research confirming its safety and efficacy in reducing perioperative hemorrhage in cardiac, obstetric, urological, and orthopedic surgeries (Colomina et al., 2017; Ker et al., 2012; Henry et al., 2001). Typically administered before or during surgery, TXA reduces perioperative blood loss (CRASH-2 trial collaborators et al., 2010). Several clinical investigations have indicated its significant impact on minimizing blood loss, decreasing postoperative transfusion rates, shortening hospital stays, and not increasing thromboembolic events (Lee et al., 2017; Kayupov et al., 2017; Zhang et al., 2017). However, the dose of tranexamic administered during the perioperative period varies widely, and there is currently no consensus on which is safer and more effective. Some studies suggest TXA’s efficacy depends on dosage, with higher doses showing safety and effectiveness in reducing hemorrhage (Hodgson et al., 2015; Johnson et al., 2017; Lin et al., 2018). Yet, direct comparisons of high-dose and low-dose TXA are limited, and the ideal perioperative period remains controversial. Therefore, this study aimed to present a narrative review of the clinical use of high-dose tranexamic acid in the perioperative period by integrating the available evidence, systematically analyzing its use in different surgical scenarios, and providing critical perspectives on the direction of future research.

**FIGURE 1:**
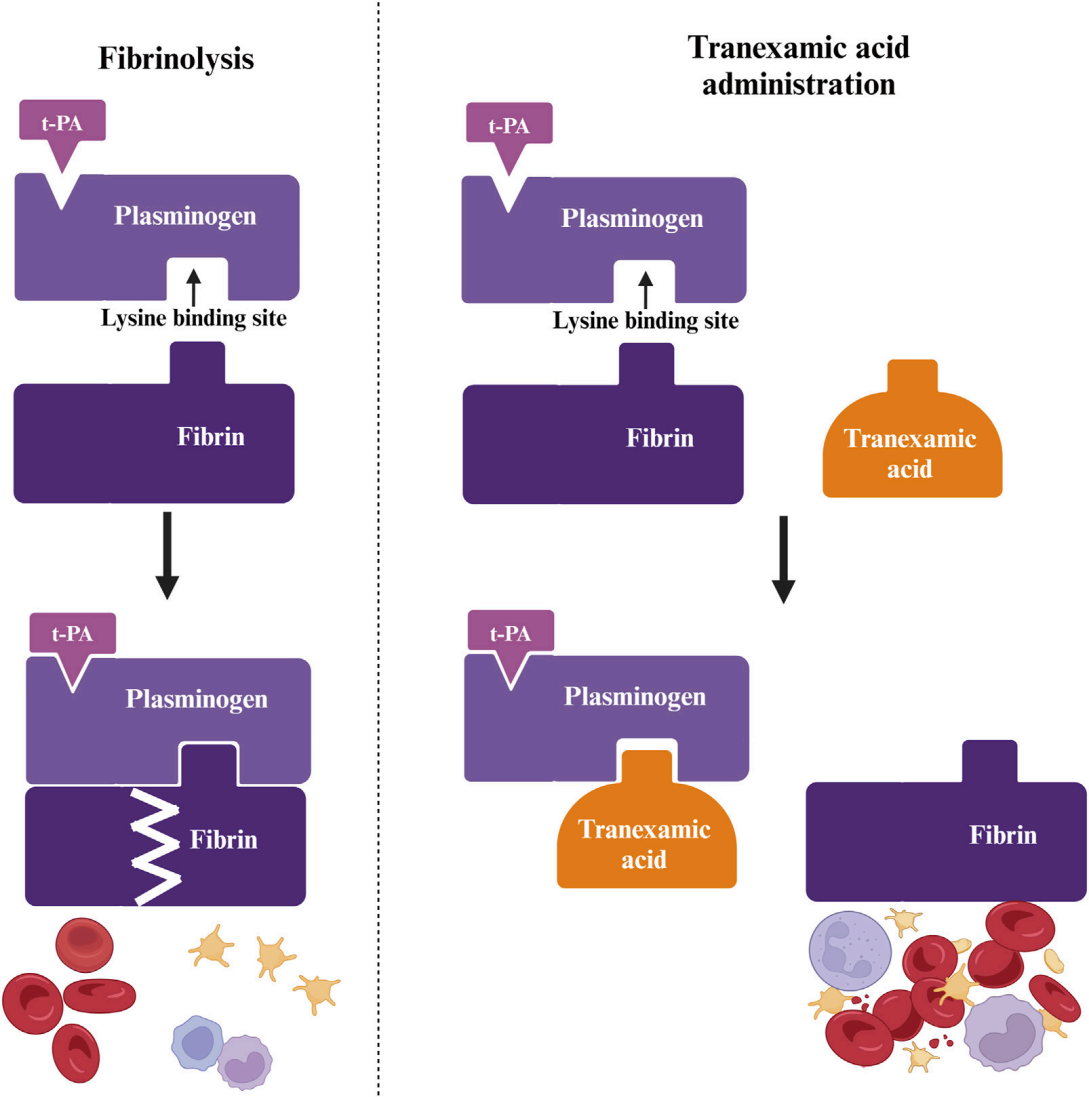
Antifibrinolytic action of tranexamic acid. Fibrinogen attaches to fibrin at the lysine binding site and is converted to fibrinolytic enzymes in the presence of tissue-type fibrinogen activator (t-PA). Tranexamic acid blocks the lysine binding site, thereby inhibiting the entry of fibrinogen into the fibrin molecule.

During hemostasis, coagulation factors activate rapidly at vascular injury sites, producing thrombin, which activates fibrinogen into stable fibrin, forming a clot. However, plasmin in blood can dissolve fibrin, compromising clot stability (Ker et al., 2012). Since plasmin can prematurely break down blood clots at bleeding sites, it plays a significant role in bleeding and rebleeding, potentially exacerbating the hemorrhage (Henry et al., 2001; CRASH-2 trial collaborators et al., 2010). Normally, plasminogen attaches to fibrin via lysine-binding sites, with tissue plasminogen activator (t-PA) initiating fibrinolysis, leading to substantial fibrin breakdown by plasmin, which may cause severe bleeding. Tranexamic acid competitively inhibits lysine binding to plasminogen, blocking the interaction between plasminogen and plasmin’s heavy chain on fibrin’s lysine residues, thereby reducing fibrin breakdown and achieving antifibrinolytic and hemostatic effects (Dunn and Goa, 1999) (Figure 1).

## 2 Materials and methods

### 2.1 Search strategy

We systematically searched PubMed, Cochrane Library, Science, Embase, and CNKI databases, from their inception to January 2025. We used the following search terms: ‘‘high-dose,” “tranexamic acid,” “perioperative,” “bleeding,” and “safety.” Search strategies were adapted for the various search engines. Manual retrieval from the references of subject-related articles was performed to broaden the search. We did not restrict our search by region or language. Two reviewers (Y.D. and X.W.) independently assessed each included study, and any discrepancies were resolved through consensus. The characteristics of the key studies are shown in Table 1.

**TABLE 1:** Summary of key studies of high-dose tranexamic acid in different types of surgery.

Author and year	Study design	Intervention	Loading dose	Maintenance dose (time)	Sample size (n)	Main results	Complication	Surgical type
Bouet (2016)	Retrospective	High-dose TXA	4 g	1 g/h	138	The difference of hemoglobin before and after delivery in High-Dose TXA group was smaller than that in control group	no deep vein thrombosis, renal function damage, seizures and maternal death	Obstetric surgery
		Blank control	—	—	151			
Shi et al. (2022)	RCT	High-dose TXA	30 mg/kg	16 mg/kg/h	1,525	The high-dose TXA group had a lower risk of receiving allogeneic red blood cell infusion	There was no difference in 30 days mortality, seizures, renal dysfunction and thrombotic events between the two groups	Cardiac surgery
		Low-Dose TXA	10 mg/kg	2 mg/kg/h	1,506			
Guo et al. (2022)	Retrospective	High-dose TXA	>50 mg/kg		196	High dose TXA significantly reduced postoperative blood loss	There was no difference in adverse events between the two groups	Cardiac surgery
		Low-dose TXA	≤50 mg/kg		196			
McHugh et al. (2016)	Retrospective	High-dose TXA	30 mg/kg	15 mg/kg/h	104	No significant difference in the infusion volume of blood products and the incidence of re examination bleeding between the two groups	There was no difference in postoperative complications and 30 days mortality between the two groups	Cardiac surgery
		Low-dose TXA	15 mg/kg	6 mg/kg/h	52			
Du et al. (2014)	RCT	High-dose TXA	30 mg/kg	16 mg/kg/h	87	No significant difference in 24-h postoperative bleeding, allogeneic blood transfusion, and mortality rate between the two groups	There was no difference in adverse events between the two groups	Cardiac surgery
		Low-dose TXA	10 mg/kg	2 mg/kg/h	88			
Samir et al. (2021)	RCT	High-dose TXA	50 mg/kg	5 mg/kg/h	95	The high-dose group can significantly reduce intraoperative blood loss	No adverse reactions were found	Prostate surgery
		Placebo	—	—	91			
Kushioka et al. (2017)	Non randomized case-control trials	High-dose TXA	2 g	2 g	30	High doses can significantly reduce intraoperative and postoperative blood loss	There were no perioperative complications	Spinal surgery
		Blank control	—	—	30			
Tumber et al. (2022)	Retrospective	High-dose TXA	≥30 mg/kg	10 mg/kg/h	126	High dose TXA is beneficial for reducing perioperative blood loss in patients	No adverse reactions occurred in both studies	Spinal surgery
		Low-dose TXA	<30 mg/kg	10 mg/kg/h	97			
Johnson et al. (2017)	Retrospective	High-dose TXA	50 mg/kg	5 mg/kg/h	44	High doses are more effective in reducing blood loss and transfusion rates in pediatric patients undergoing surgery for idiopathic scoliosis	Neither group experienced thrombosis or ischemic events	Spinal surgery
		Low-dose TXA	10 mg/kg	1 mg/kg/h	72			
Shrestha et al. (2021)	Meta-analysis	—	—	—	—	The high-dose intravenous group reduced blood loss during surgery for adolescent idiopathic scoliosis	High doses will not cause any significant thromboembolic events	Spinal surgery
Zhang et al. (2022)	RCT	High-dose TXA	50 mg/kg		39	Intravenous application of TXA during surgery can significantly reduce postoperative blood loss in a dose-dependent manner, and can also lower postoperative fibrinolysis indicators	High dose TXA does not increase the risk of complications such as lower limb deep vein thrombosis and pulmonary embolism	Spinal surgery
		Low-dose TXA	20 mg/kg		39			
		Placebo			38			
Lin et al. (2018)	Retrospective case analysis	High-dose TXA	50 mg/kg	5 mg/kg/h	100	High dose TXA can reduce intraoperative blood loss	Three cases of thromboembolic complications occurred. There are no cases of myocardial infarction, epilepsy, stroke, or acute kidney failure	Spinal surgery
Lei et al. (2020)	RCT	High-dose TXA	60 mg/kg	Administer 1 g in divided doses after 3, 6, 12, 18, and 24 h	66	High dose TXA can reduce perioperative blood loss and transfusion rate	High doses will not cause any significant thromboembolic events	Joint surgery
		Low-dose TXA	20 mg/kg		66			

RCT, randomized controlled trials; TXA, tranexamic acid.

### 2.2 Inclusion/exclusion criteria

The inclusion criteria were as follows: (1) clinical studies exploring high-dose tranexamic acid; (2) major types of surgical procedures involving obstetrics, cardiology, and orthopedics; and (3) peer-reviewed literature in English and Chinese. The exclusion criteria were as follows: case reports, animal studies, and low-quality studies. Literature selection focused on the comprehensiveness of subject coverage and the diversity of study designs.

## 3 Application of high-dose tranexamic acid in the perioperative period

### 3.1 Obstetric surgery

Postpartum hemorrhage continues to be a predominant factor in early maternal mortality, accounting for approximately 300,000 deaths globally each year (Waterstone, 2001; Hogan et al., 2010). A randomized, controlled multicenter study by Ducloy-Bouthors et al. found that high-dose TXA reduces blood loss in ongoing postpartum hemorrhage. In this study, patients with blood loss exceeding 800 mL after vaginal delivery were randomly assigned to receive either TXA (initial 4 g dose in the first hour, followed by a continuous infusion of 1 g/h for 6 h) or the control group. Results showed that TXA reduced hemorrhage duration and lowered the likelihood of severe postpartum hemorrhage requiring transfusion, with no severe adverse reactions noted (Ducloy-Bouthors et al., 2011). However, a later study by Bouet et al. using the same high-dose TXA protocol during vaginal delivery did not significantly reduce blood loss, though hemoglobin levels were higher post-delivery in the TXA group, and no serious adverse reactions (e.g., renal impairment, seizures, or maternal death) were reported (Bouet et al., 2016). The discrepancies between the different research results may be attributed to variations in the study design, implementation details, and statistical methods. As adverse events are comparatively infrequent, more extensive international studies are required to inform the use of high-dose TXA in postpartum hemorrhage.

### 3.2 Cardiac surgery

Extensive research has shown that tranexamic acid significantly reduces postoperative blood loss, the need for blood transfusions, and reoperation rates in cardiac surgery, leading to its recommendation in such procedures (Guo et al., 2022; Brown et al., 1997). Nonetheless, the optimal dosage of tranexamic acid for cardiac surgery remains debatable. In a multicenter, randomized, double-blind study involving patients scheduled for cardiopulmonary bypass surgery, participants were allocated to either a high-dose tranexamic acid group (initial dosage of 30 mg/kg, sustaining dosage of 16 mg/kg/h) or a low-dose tranexamic acid group (initial dosage of 10 mg/kg, sustaining dosage of 2 mg/kg/h). Research has indicated that individuals in the high-dose cohort had a lower likelihood of undergoing allogeneic red blood cell transfusions, with a statistically significant difference compared to the low-dose group. Furthermore, there were no statistically significant differences between the two groups in adverse events such as seizures, renal failure, thrombotic incidents, and overall mortality (Shi et al., 2022). Additionally, a retrospective cohort study by Guo et al. assessed postoperative blood loss over 3 days for patients undergoing acute type A aortic dissection surgery. The study compared outcomes between patients who received high-dose tranexamic acid (>50 mg/kg) and those who received low-doses (≤50 mg/kg), finding that the high-dose group experienced significantly less postoperative blood loss. Moreover, there were no notable differences in the occurrence of thromboembolic incidents (including myocardial infarction, pulmonary embolism, deep vein thrombosis, and stroke) or seizures between the groups (Guo et al., 2022). Conversely, a retrospective study by McHugh et al. involving patients undergoing coronary artery bypass grafting found no significant difference in the amount of blood transfusions or frequency of additional bleeding investigations between those treated with high-dose tranexamic acid (initial dosage of 30 mg/kg, sustaining dosage of 15 mg/kg/h) and those treated with low-dose tranexamic acid (initial dosage: 15 mg/kg, sustaining dosage: 6 mg/kg/h). Nevertheless, the postoperative complication and mortality rates were comparable between the two groups (Subramaniam et al., 2016). Studies conducted by Du et al. revealed that among patients undergoing heart valve surgery reported no significant differences in postoperative bleeding or allogeneic blood product use between high- and low-dose groups (initial dosage of 30 mg/kg, sustaining dosage of 16 mg/kg/h; 10 mg/kg initial dose, 2 mg/kg/h for low-dose) (Du et al., 2014). Despite these findings, some studies have suggested a potential link between tranexamic acid and neurological complications, particularly generalized tonic-clonic seizures, following cardiopulmonary bypass (Martin et al., 2008). Using multivariable logistic regression models, Kalavrouziotis et al. discovered that high-dose tranexamic acid (total dose >100 mg/kg during the perioperative phase) was independently associated with an increased risk of early seizures (Kalavrouziotis et al., 2012). Nevertheless, this retrospective study had several limitations. Additionally, variations in study outcomes may arise due to different surgical techniques, thereby necessitating more extensive clinical trials to evaluate the effectiveness and safety of administering high doses of tranexamic acid to control bleeding after heart surgery.

### 3.3 Prostate surgery

Research has shown that 90% of men aged >70 years suffer from prostate hyperplasia, which can lead to diminished urinary tract symptoms (Samir et al., 2019). Transurethral resection of the prostate (TURP) is considered the “benchmark” treatment for prostate hyperplasia (Jendoubi et al., 2017). However, bleeding is the most common complication of transurethral prostate resection. This complication may be linked to elevated urokinase activity released by the prostate and the high concentrations of plasminogen activators in the urine and urinary epithelium that activate the fibrinolytic system (Miernik and Gratzke, 2020; NIELSEN et al., 1997; Rannikko et al., 2004). Administering TXA during prostate surgery has been shown to reduce blood loss effectively. However, the optimal dosage of TXA remains unclear. Many current studies suggest that using TXA during surgery can significantly reduce blood loss in prostate procedures (Rannikko et al., 2004; Longo et al., 2018; Vanderbruggen et al., 2023). A contemporary prospective randomized controlled trial by Samir et al. randomly assigned 204 patients with prostate hyperplasia to either a high-dose TXA group (initial dosage: 50 mg/kg, sustained dosage: 5 mg/kg/h) or a control group (saline only). The findings indicated that high-dose TXA markedly diminished blood loss during the intraoperative period, improved surgical conditions, shortened operation time, and reduced the volume of irrigation fluid used during surgery. Importantly, no adverse events - such as acute kidney injury, convulsions, pulmonary embolism, or heart attack were observed (Samir et al., 2021). Nevertheless, owing to the limited number of participants in this study and the absence of TXA in the control group, assessing the clinical effects of different tranexamic acid dosages in prostate surgery was unfeasible. Therefore, more extensive studies with larger sample sizes are required to guide the application of tranexamic acid in prostate surgery.

### 3.4 Spinal surgery

Spinal surgeries carry a high risk of blood loss, making perioperative blood management crucial. TXA has shown promise in reducing intraoperative and perioperative blood loss in these procedures (Ferraris et al., 2012). A meta-analysis by Gill et al. reviewed seven studies and concluded that TXA significantly reduced the need for blood loss transfusions in spinal surgery (Gill et al., 2008). In recent years, numerous studies have demonstrated that high-dose tranexamic acid is both effective and safe for spinal surgery. Kushioka et al. evaluated the hemorrhage in individuals undergoing posterior lumbar interbody fusion, and the findings indicated that the cohort receiving high-dose tranexamic acid (preoperative: 2000 mg, postoperative 16 h: 2,000 mg) had significantly reduced intraoperative and postoperative hemorrhage compared to the control group (Kushioka et al., 2017). Tumber et al. retrospective analyzed 223 adolescent idiopathic scoliosis patients, finding that a high-dose TXA regimen (initial dosage: ≥30 mg/kg, sustaining dosage: 10 mg/kg/h) was more effective in reducing blood loss than low-dose regimen (initial dosage of <30 mg/kg, sustaining dosage of 10 mg/kg/h). The results of this study indicated that a high dose of TXA markedly reduced blood loss during surgical procedures (Tumber et al., 2022). Similarly, research conducted by Johnson et al. demonstrated that in pediatric scoliosis surgical procedures, patients receiving high-dose tranexamic acid (initial dose of 50 mg/kg, sustained dose of 5 mg/kg/h) exhibited lower blood loss than those administered low-dose tranexamic acid (initial dose of 10 mg/kg, sustained dose of 1 mg/kg/h) (Johnson et al., 2017). Furthermore, an extensive meta-analysis involving 334 patients confirmed the efficacy of high-dose TXA in reducing blood loss and shortening the surgical period for adolescent idiopathic scoliosis treatments (Shrestha et al., 2021). Nonetheless, the dosing protocols for TXA in the aforementioned investigations were not derived from pharmacokinetic principles, leading to considerable debate regarding the application of high-dose TXA. A randomized double-blind study conducted by Hasan et al. demonstrated that among participants aged 10–21 years diagnosed with adolescent idiopathic scoliosis and slated for elective single-stage posterior spinal fusion surgery, the administration of high-dose tranexamic acid (initial dosage of 30 mg/kg, sustained dosage of 10 mg/kg/h) did not result in a significant reduction in intraoperative blood loss in comparison to the low-dose TXA group (initial dosage of 10 mg/kg, sustained dosage of 1 mg/kg/h). Additionally, there was no notable difference in the need for allogeneic blood transfusion between the two cohorts (Hasan et al., 2021). Numerous studies have validated the safety of administering high-dose TXA during spinal surgical procedures (Zhang et al., 2022). Brown et al. performed a retrospective study that included 36 patients who underwent spinal surgery and found that none of the patients treated with high-dose TXA during their operations experienced thromboembolic events or any significant complications (Brown et al., 2023). In the investigation by Lin et al., aimed at assessing the safety of a high-dose TXA regimen (initial dosage of 50 mg/kg, sustaining dosage of 5 mg/kg/h), out of 100 patients undergoing spinal deformity correction with high-dose TXA three instances of thromboembolic complications were documented. This encompassed one instance of pulmonary embolism and two occurrences of deep vein thrombosis, all of which were effectively managed with anticoagulation therapy. No cases of myocardial infarction, seizures, stroke, or acute renal failure have been reported (Lin et al., 2018). Moreover, pertinent meta-analyses have established that high-dose tranexamic acid regimens are both safe and effective in reducing intraoperative bleeding during spinal surgeries (Akosman et al., 2023). Nevertheless, it remains imperative to exercise caution when monitoring patients receiving high-dose tranexamic acid to prevent any associated complications.

### 3.5 Joint surgery

Joint replacement surgery, especially in severe joint diseases, often induces a high fibrinolytic state, which can lead to intraoperative and postoperative blood loss, resulting in postoperative anemia, which may increase the mortality rate after joint replacement surgery (Xie et al., 2016; Lei et al., 2017; Rineau et al., 2016). Therefore, the significance of TXA in arthroplasty has gradually become apparent. Hiippala et al. were the pioneers in elucidating the advantages of using TXA in knee replacement surgery (Hiippala et al., 1995). A prospective investigation conducted by Akgül et al. demonstrated that a single administration of tranexamic acid via an intravenous route at a high dose of 20 mg/kg markedly decreased the volume of blood loss during surgery and within 24 h postoperatively. Previous studies have shown that compared to a single administration of TXA via the intravenous route, the utilization of TXA in multiple perioperative contexts during total hip arthroplasty has been shown to substantially reduce blood loss during the perioperative period, decrease the need for blood transfusions, and impede fibrinolysis without contributing to an elevated risk of thromboembolic events (Lei et al., 2018; Lin and Xiaoyi, 2016). A prospective randomized controlled trial by Lei et al. compared high-dose (60 mg/kg initial dose, maintenance of 1 g at intervals of 3, 6, 12, 18, and 24 h). The high-dose regimen was more effective in reducing perioperative blood loss and transfusion needs (Lei et al., 2020). Similarly, Cui et al., demonstrated that high-dose TXA reduced blood loss in unilateral total hip arthroplasty without any documented thromboembolic events.

## 4 Conclusion

Perioperative bleeding is closely related to adverse outcomes in patients, and significant perioperative blood loss can lead to serious adverse events. TXA, an antifibrinolytic agent, is gradually gaining prominence in perioperative settings. However, the optimal dosage of TXA still needs to balance the clinical benefits and risks of different surgical sites and procedures. Currently, high-dose TXA has been applied in obstetrics, cardiovascular surgery, urology, and orthopedics-particularly in cardiac and spinal surgeries - with extensive research indicating that it significantly reduces perioperative blood loss without severe adverse effects. The application of elevated doses of TXA should not be universal but should demonstrate its advantages for surgical sites and procedures associated with an increased likelihood of hemorrhage. Therefore, large-scale, multicenter randomized controlled trials are necessary to establish clear guidelines for the perioperative period use of high-dose TXA.
